# Interfacial Properties and Hopping Diffusion of Small Nanoparticle in Polymer/Nanoparticle Composite with Attractive Interaction on Side Group

**DOI:** 10.3390/polym10060598

**Published:** 2018-05-29

**Authors:** Kai-Xin Ren, Xiang-Meng Jia, Gui-Sheng Jiao, Tao Chen, Hu-Jun Qian, Zhong-Yuan Lu

**Affiliations:** State Key Laboratory of Supramolecular Structure and Materials, and Laboratory of Theoretical and Computational Chemistry, Institute of Theoretical Chemistry, Jilin University, Changchun 130023, China; renkx15@mails.jlu.edu.cn (K.-X.R.); jiaxm14@mails.jlu.edu.cn (X.-M.J.); jiaogs@ihep.ac.cn (G.-S.J.); chentao314@163.com (T.C.); luzhy@jlu.edu.cn (Z.-Y.L.)

**Keywords:** polymer/nanoparticle composite, nanoparticle diffusion, interfacial structure and dynamics

## Abstract

The diffusion dynamics of fullerene (C${}_{60}$) in unentangled linear atactic polystyrene (PS) and polypropylene (PP) melts and the structure and dynamic properties of polymers in interface area are investigated by performing all-atom molecular dynamics simulations. The comparison of the results in two systems emphasises the influence of local interactions exerted by polymer side group on the diffusion dynamics of the nanoparticle. In the normal diffusive regime at long time scales, the displacement distribution function (DDF) follows a Gaussian distribution in PP system, indicating a normal diffusion of C${}_{60}$. However, we observe multiple peaks in the DDF curve for C${}_{60}$ diffusing in PS melt, which indicates a diffusion mechanism of hopping of C${}_{60}$. The attractive interaction between C${}_{60}$ and phenyl ring side groups are found to be responsible for the observed hopping diffusion. In addition, we find that the C${}_{60}$ is dynamically coupled with a subsection of a tetramer on PS chain, which has a similar size with C${}_{60}$. The phenyl ring on PS chain backbone tends to have a parallel configuration in the vicinity of C${}_{60}$ surface, therefore neighbouring phenyl rings can form chelation effect on the C${}_{60}$ surface. Consequently, the rotational dynamics of phenyl ring and the translational diffusion of styrene monomers are found to be slowed down in this interface area. We hope our results can be helpful for understanding of the influence of the local interactions on the nanoparticle diffusion dynamics and interfacial properties in polymer/nanoparticle composites.

## 1. Introduction

It is well known that adding nanoparticles (NPs) into polymer material can often dramatically alter the associated structure [1,2,3], dynamics [4,5,6] and mechanical [6,7] properties. However, such property alternations resulted from addition of NPs depend on many factors, e.g., interactions between polymer and NP, molecular weight and chain topology of polymer, size and shape of NP, etc. At the same time, these factors are always intercorrelated and they differ between different systems. Therefore, microscopic mechanisms controlling polymer/NP composite (PNC) material properties are expected to vary between systems and are far away from fully explored.

For instance, as a model NP with well-defined chemical structure and monodisperse particle size, C${}_{60}$ has been used in many experimental studies. Several of these studies [8,9,10] have demonstrated that adding C${}_{60}$ NP into polystyrene matrix where NP has a favourable attractive interaction with polymer can result in an increase in glass transition temperature, $T_{g}$. Such increase in $T_{g}$ is also observed in composite systems of C${}_{60}$/poly(methyl methacrylate) [8,11] and C${}_{60}$/tetramethylbisphenol-A polycarbonate [8]. As demonstrated by MD simulations, such increase in $T_{g}$ can be attributed to the attractive interactions between NP and polymer, which can decrease the mobility of polymer segments surrounding the NP. On the contrary, these simulation results also demonstrate that repulsive interaction between NP and polymer can decrease $T_{g}$. A direct evidence can be found in the composite system of polypropylene/C${}_{60}$, where a decrease in $T_{g}$ of 14.5 K is found after dispersing C${}_{60}$ NP in the system [12]. At the same time, it was also reported that addition of C${}_{60}$ into PP matrix will decrease the elongation to break, the energy to break and the tensile strength of the material during a tensile pull test [12]. However, influence of C${}_{60}$ on polymer melt viscosity is much more complicated. Tuteja [13] reported that adding C${}_{60}$ NPs will result in an increment in melt viscosity of unentangled linear PS melt, whereas in entangled melts it presents a viscosity reduction effect. For the star PS melts, Tang and coworkers [5] reported a decrease in viscosity for short chain length system and an increase in viscosity for entangled long arm system after adding C${}_{60}$ NPs. A recent molecular dynamics (MD) simulation study [14] also shows that polymers with different chain architectures will have different dynamic coupling with embedded NPs and therefore different influences on the NP motion. Therefore, we still have a long way to go before we can fully explore the complex influence mechanisms of these NPs on the material properties. Among many efforts, a full understanding of diffusion dynamics of NPs [15,16,17] and their influences on the structure and dynamic properties of the surrounding polymer segments is undoubtedly crucial.

In general, it has been widely realised that changes in macroscopic properties are directly related to the formation of interfacial layer where both structure and dynamics properties of polymers are different from the bulk due to the presence of the NPs. Thickness of such interfacial layer may depend on characteristics of both NP and polymer, i.e., size, shape and softness of the NP [18], and chain rigidity [19] and molecular weight (MW) [20] of the polymer chain. For instance, after performing both extensive full atomistic and coarse-grained molecular dynamics (MD) simulation, Müller-Plathe and coworkers [21,22,23] found that polystyrene (PS) chains can have a layer structure and a reduced dynamics around the SiO${}_{2}$ NP surface. The thickness of the interface area, i.e., the influence range of the NP, can vary from 1 to 3 radii of gyration ($R_{g}$) of matrix PS chain, depending on the properties investigated. Range of NP influence on the dynamics properties were found to be much larger than that on the chain structures. Experimental measurements [24,25,26] also suggest that thickness of the adsorbed polymer layer on NP surface increases with an increase in MW of the polymer and is proportional to $R_{g}$ of polymer. Recent experimental measurements by Sokolov and coworkers [27] even revealed that hydrogen bonding interactions between SiO${}_{2}$ NP and poly(2-vinylpyridine) chains can be influenced by the MW of the polymer. Note that NPs in these works are usually SiO${}_{2}$ which are at least several nanometers in diameter. Interestingly, a recent MD simulation work by Vogiatzis and Theodorou [28] reported that presence of small C${}_{60}$ NPs can also suppress the surrounding segment dynamics of polystyrene chains. Another work reported by Cheng and coworkers [29] demonstrated that well-dispersed small NPs (<2 nm in diameter) with attractive interactions with matrix polymers can lead to unexpectedly large and very different changes in PNC dynamics in comparison to conventional PNC materials containing relatively larger SiO${}_{2}$ NPs.

For the diffusion dynamics of NPs, extensive theoretical works reported in the literature [30,31,32,33,34,35,36] predicted that its mobility will deviate from the Stokes–Einstein relation and there will be a length scale dependent viscosity effect when NP diffuses in a polymer melt. In particular, Schweizer and coworkers developed a force-level generalised Langevin Equation (GLE) approach to study the mobility of a single NP in unentangled and entangled polymer melts. In unentangled melts, the NP motion is determined by the polymer constraint-release mechanism, while in entangled melts, the NP mobility is mainly related to the ratio of the NP radius ($R_{a}$) and the tube diameter ($d_{T}$) [31,33]. Similarly, according to the scaling theory developed by Cai et al. [35], diffusion of small particles with $R_{a}\leq \xi$ (ξ is correlation length of the polymer melt) does not depend on the structural relaxation of the matrix polymers. For intermediate sized NP with $\xi \leq R_{a}\leq d_{T}$, its diffusion is mainly affected by the relaxation of the polymer segments with a similar size of NP ($R_{g}^{\mathrm{seg}}∼R_{a}$), where $R_{g}^{\mathrm{seg}}$ is the radius gyration of the polymer segment. For large NPs with $R_{a}>d_{T}$, the diffusion is largely controlled by the entanglement mesh. These theoretical predictions have also been confirmed by many experiments [37,38,39] and simulations [40,41,42]. A recent simulation [43] even shows that the polydispersity in NP size can have non-negligible impacts on the diffusion dynamics of NPs in polymer melts.

Cai et al. [36] predicted that the terminal diffusion of the NP is dominated by a hopping motion when the NP has an intermediate size that is moderately larger than $d_{T}$. For much larger NPs, they will be trapped by local networks but they can still move in polymer melts by waiting for entanglement meshes to relax [36]. Moreover, Schweizer and co-workers also put forward an activated hopping mechanism of NPs in cross-linked networks and entangled melts based on their nonlinear Langevin equation theory [32]. According to these theories, NP hopping is relevant only for particle with size $2R/d_{T}∼$ 1.5–2, while smaller particles are considered to slip through the entanglement mesh [32,36]. It is also worth noting that there is no evidence for hopping motion of NPs in simulations based on coarse-grained bead-spring models [42]. However, very recently, Volgin et al. [44] found that in polymide (PI) matrix small C${}_{60}$ NP also adopted hopping diffusion, where C${}_{60}$ NP only has a small size ratio of $2R/d_{T}∼0.1$ with respect to the tube diameter ($d_{T}$). This phenomenon is obviously beyond the above theoretical predictions [32,36]. The hopping diffusion of small C${}_{60}$ NP in PI matrix can be attributed to the attractive interactions between C${}_{60}$ and the polymer chain backbone, C${}_{60}$ was found to dynamically coupled to the translational motion of a flat segment commensurate with the C${}_{60}$ size. Indeed, such particular features of the “polymer–nanoparticle” interactions can significantly affect the properties of PNC materials [40,45,46,47]. However, due to rich variety of polymers, how the interactions between NPs and polymer segments especially the side groups of polymer influence the NP dynamics remain largely unexplored.

In this article, we perform all-atom molecular dynamic simulations of two model composite systems composed of C${}_{60}$ NP and two different short unentangled polyvinyl polymers, namely PS and PP, where C${}_{60}$ NP has a diameter of only 0.7 nm and is much smaller than traditional SiO${}_{2}$ NP. From the computational point of view, such a small NP has a fast diffusion and therefore ensures that the NP diffusion dynamics can be accessible for the full atomistic simulations with limited computational resources. In the composite system of PS/C${}_{60}$, there are attractive interactions between C${}_{60}$ and phenyl rings on side group of PS chain, while such interactions are absent in the system of PP/C${}_{60}$. Local structure and dynamics of polymer segments at interface area in the vicinity of the NP surface and the diffusion dynamics of NP are investigated. In particular, we find a hopping diffusion process of C${}_{60}$ in PS melt due to attractive interactions between C${}_{60}$ and phenyl ring side groups. We also find a dynamic coupling between C${}_{60}$ and a PS tetramer which has a similar size with NP. Phenyl rings on the PS chain backbone are found to be parallel to the surface of C${}_{60}$ NP in its vicinity. At the same time, rotational dynamics of phenyl ring and translational diffusion of styrene monomer are found to be slowed down in this interface area. The simulation details are given in Section 2. In Section 3, all simulation results are given. Finally, the main conclusions are summarised in Section 4.

## 2. Materials and Simulation Methods

In our simulation, both PS/C${}_{60}$ and PP/C${}_{60}$ composite systems have a polymer chain length of *N* = 20. Production run for both systems are performed at *T* = 500 K. Since one of our purposes is to make a comparative study of the dynamics of C${}_{60}$ in two composites, two systems with similar equilibration box size are designed, as listed in Table 1. There are 40 and 70 polymer chains in PS and PP systems, respectively. Note that, to avoid the influence of the interactions between C${}_{60}$ NPs, we only consider one C${}_{60}$ NP in each simulation system. In our simulation, the all-atom OPLS-AA force field parameters [48,49] are employed in our simulations, with this model all hydrogen atoms are explicitly treated in our simulation. Due to a slow relaxation and therefore a hard equilibration of such all-atom systems, a careful equilibration process is used to equilibrate the initial simulation system, details of this process can be found in the next paragraph. After the equilibration, we perform a long NPT run up to 3 μs under an atmospheric pressure at 500 K for both systems. The Nosé–Hoover [50,51] thermostat with a coupling time of 0.2 ps and Parrinello–Rahman [52] barostat with a coupling time of 1 ps are used to control the temperature and pressure, respectively. The leap-frog algorithm is used to integrate the Newton’s equations of motion with a time step of 1 fs for all the simulations. All full atomistic MD simulations are performed using GROMACS 5.1 [53].

The construction and equilibration of the initial simulation box is performed in the following procedure: (i) For PS chains, fully stretched (all trans-) PS chains are firstly constructed by replicating two different types of styrene units along the chain backbone, that have *R* or *S* configurations according to the IUPAC definition. In this step, a uniform random number located in (0.0,1.0) is used to control the stereochemistry of the chain. A random number greater than 0.5 gives a *R* configuration and otherwise a *S* configuration, therefore the system has a monomer ratio of R/S = 1:1. For the PP chains, the initial construction of the chain follows the same procedure as PS. The orientation of the methyl group is controlled by the random number, half of the methyl groups are set on the left side of the backbone while half of them are on the right. (ii) After construction, these fully stretched chains are relaxed in a vacuum condition under a short canonical ensemble (NVT) run for 10 ns at a relatively high temperature *T* = 900 K. (iii) The pre-relaxed chain configurations are subsequently packed into the simulation box with the PACKMOL program [54] with a low initial density of 0.041 g/cm${}^{3}$ for PS system and 0.012 g/cm${}^{3}$ for PP system. The C${}_{60}$ is also packed into the box in this step. The initial cubic simulation box side length is set at 15 nm for PS and 20 nm for PP system. For a fast equilibration, a short 10 ns NVT run and subsequently a 200 ns isothermal-isobaric ensemble (NPT) run are performed to eliminate local unreasonable stress and to attain an equilibrated density, temperature is set at 900 K for PS system and 700 K for PP system in this step. It results a equilibrium density of 0.644 g/cm${}^{3}$ and 0.556 g/cm${}^{3}$ for PS/C${}_{60}$ composite at 900 K and PP/C${}_{60}$ composite at 700 K, respectively. Note that, although the temperature is rather high and the system density is rather low, the system is already in a condensed phase. (iv) Starting from the configuration obtained in the last step, designed repeated annealing cycles are performed either between 900 K and 500 K for PS/C${}_{60}$ system or between 700 K and 500 K for PP/C${}_{60}$ system. The PS/C${}_{60}$ system is cooled down from 900 K to 500 K and then heated up back to 900 K, and finally once again cooled down to 500 K. The heating/cooling rate we used is ±0.004 K/ps. For the PP/C${}_{60}$ system, it is cooled down from 700 K to 500 K and then heated up back to 700 K, and finally once again cooled down to 500 K. The heating/cooling rate is ±0.0032 K/ps. After the above annealing cycle, an additional 50 ns NPT run was performed for both systems. In the above equilibration process, Berendsen thermostat and barostat are used to control the system temperature and pressure. The coupling time used is 0.2 ps and 1 ps for thermostat and barostat, respectively.

Note that, since C${}_{60}$ has a very small diameter (∼0.7 nm) which is much smaller than $d_{T}$, we believe it is not necessary to simulate long enough entangled polymers. According to theoretical work by Cai et al. [35], diffusion dynamics of C${}_{60}$ will be mainly affected by the relaxation of the polymer segments with a similar size of NP. Indeed, we find a dynamic coupling between C${}_{60}$ and PS tetramer as reported below. In addition, one has to use less detailed coarse-grained models for really entangled long polymers for the purpose of computational efficiency, however a recent study using such a model [42] did not observe any hopping of NP. Therefore, we only simulate short unentangled polymers in our simulation.

**Figure 1:**
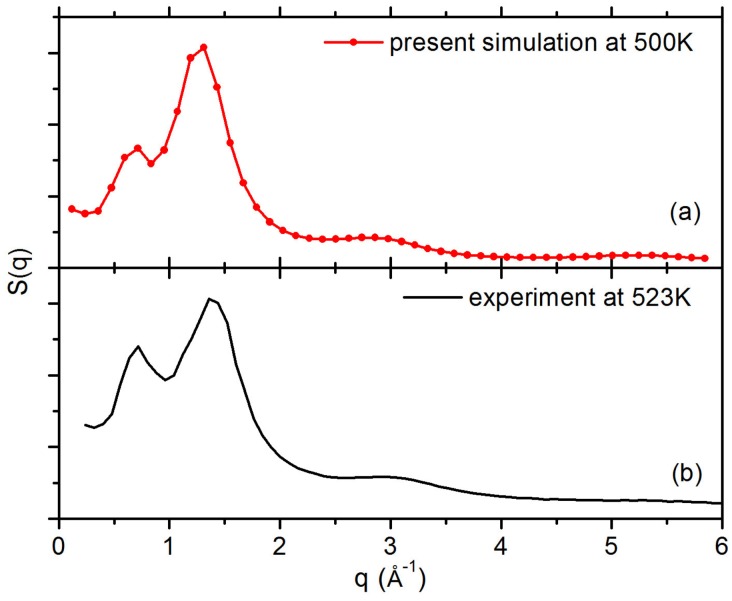
The static structure factors S(*q*) of PS melt (**a**) calculated from simulation at 500K; and (**b**) from experimental X-ray diffraction reported in Ref. [60] at 523 K. Data in (**b**) are reprinted from Ref. [60] with permission from Elsevier.

For both PS and PP systems, the last 2.5 μs trajectory is used to analyse the data. Results of the mass density, radius of gyration ($R_{g}$) and end-to-end distance ($R_{\mathrm{ee}}$) for polymers are listed in Table 1 for both systems. For PS system, the density ρ = 0.952 g/cm${}^{3}$, the radius of gyration ${\langle R_{g}^{2}\rangle }^{1/2}=0.982$ nm and the end-to-end distance ${\langle R_{\mathrm{ee}}^{2}\rangle }^{1/2}=2.566$ nm are in good agreement with other simulations [55,56] and experiments [57]. For the PP system, a density value of 0.783 g/cm${}^{3}$ is obtained from an additional simulation at 400 K. Although the value is a little lower than the value of 0.802 g/cm${}^{3}$ given by Orwoll [58] for atactic PP at 393 K, the agreement is still quite good considering the temperature difference and the fact that the chain length in our simulation is relatively short. Moreover, we obtain the relaxation time $\tau_{\mathrm{KWW}}$ of PS and PP chains by fitting a Kohlrausch–Williams–Watts (KWW) function, and the details are shown in Figure S1 in Supplementary Materials (SM). The results of $\tau_{\mathrm{KWW}}$ are listed in Table 1. To further verify our models, we have calculated the static structure factors S(*q*) of PS melt to characterise all carbon-carbon correlations, as shown in Figure 1a. Our results are in good agreement with other simulations [59] and experimental X-ray diffraction data from Ref. [60] at 523 K as shown in Figure 1b. There are two well-defined peaks, the first peak appears at about 0.6 ${Å}^{-1}$ which is called “polymerisation peak”, and the second peak is located at 1.3 ${Å}^{-1}$ which is related to segmental correlations between the neighbouring chains.

## 3. Results and Discussion

### 3.1. Interfacial Structure and Dynamics

**Figure 2:**
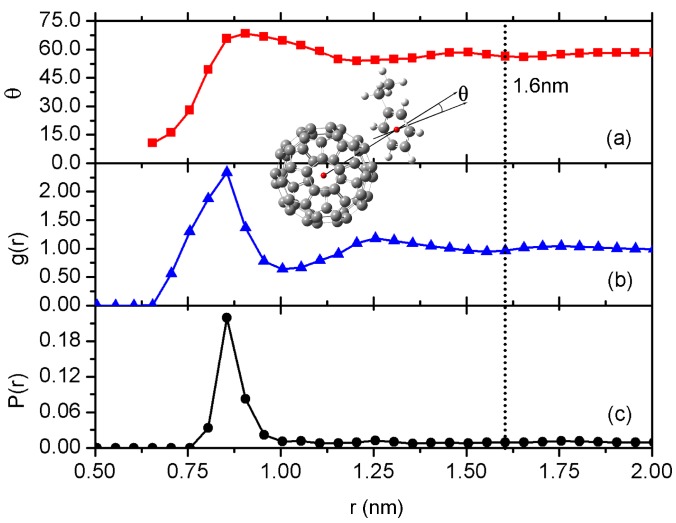
(**a**) The orientation angle of phenyl rings around C${}_{60}$; (**b**) the RDF of phenyl rings on melt PS chain backbone around the C${}_{60}$ NP surface; and (**c**) the distribution of paralleled phenyl ring pairs around C${}_{60}$. Note that all the data in these figures are plotted as a function of the distance to the centre-of-mass of the C${}_{60}$ NP. The inset shows the definition of the orientation angle of the phenyl ring.

As discussed in the above section, there are π−π interactions between C${}_{60}$ and phenyl rings on PS chain, as evidenced from density functional theory calculations performed in Ref. [61], while it is absent in PP system. It is also consistent with experimentally reported $T_{g}$ values, adding $C_{60}$ cause an increase in $T_{g}$ for PS [8,9,10] while a decrease in $T_{g}$ for PP [12]. To quantitatively characterise the influence of such interaction between C${}_{60}$ and PS, we calculate the orientation angle of phenyl rings in the vicinity of C${}_{60}$; the results are shown in Figure 2a. The orientation angle θ is defined between the normal vector of phenyl ring and the vector connecting the geometric centres of phenyl ring and C${}_{60}$, as depicted in the inset of Figure 2. $\theta =0^{\circ }$ corresponds to a parallel configuration of the phenyl ring on the C${}_{60}$ surface, while $\theta =90^{\circ }$ for a vertical alignment of the phenyl ring on the top of the C${}_{60}$ surface. At the vicinity of the C${}_{60}$ surface, θ angle is clearly close to $10^{\circ }$, which indicates parallel alignment of the phenyl rings on the C${}_{60}$ NP surface. When the distance becomes larger, the orientation angle gradually increases. It has a maximum angle of $\theta ∼70^{\circ }$ at a distance r∼0.875. This maximum peak corresponds to a relatively perpendicular configuration of the phenyl ring to the C${}_{60}$ surface. Such perpendicular orientation will induce a close packing of styrene monomers (phenyl rings) at this distance, as evidenced by a maximum distribution of phenyl rings at this distance around C${}_{60}$, as shown in the radius distribution function (RDF) of phenyl rings around C${}_{60}$ (see Figure 2b). To further characterise such close packing of phenyl rings at this distance, we have calculated the distribution of parallel phenyl ring pairs around C${}_{60}$ surface; the results are shown in Figure 2c. Here, parallel phenyl ring pairs have an inter phenyl ring distance of <0.4 nm and the angle between their normal vectors is smaller than $20^{\circ }$. Obviously, there is a peak at the distance r∼0.875, indicating a close packing of phenyl rings at this distance. For the phenyl rings far away from C${}_{60}$ surface, i.e., r> 1.6 nm, as indicated by the vertical dotted line in Figure 2, they present an average orientation angle of $57.3^{\circ }$ [18].

**Figure 3:**
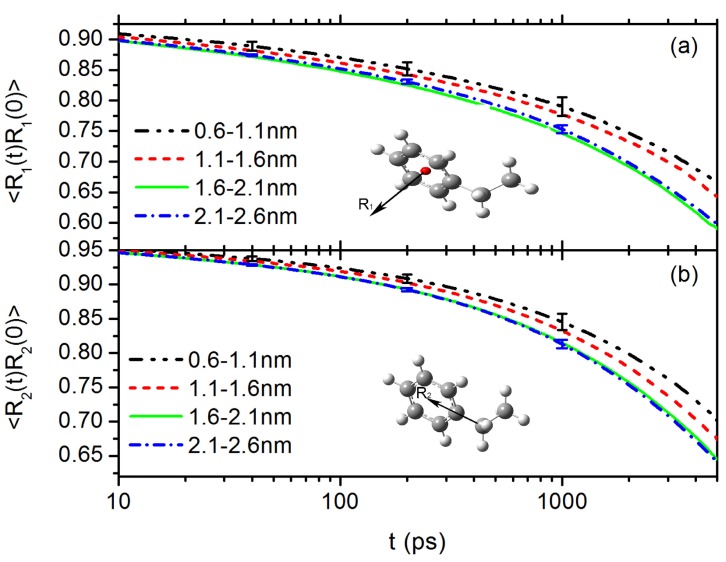
Time auto-correlation function of: (**a**) the normal vector of the surrounding phenyl rings; and (**b**) the carbon bond connecting phenyl ring and the PS chain backbone.

**Figure 4:**
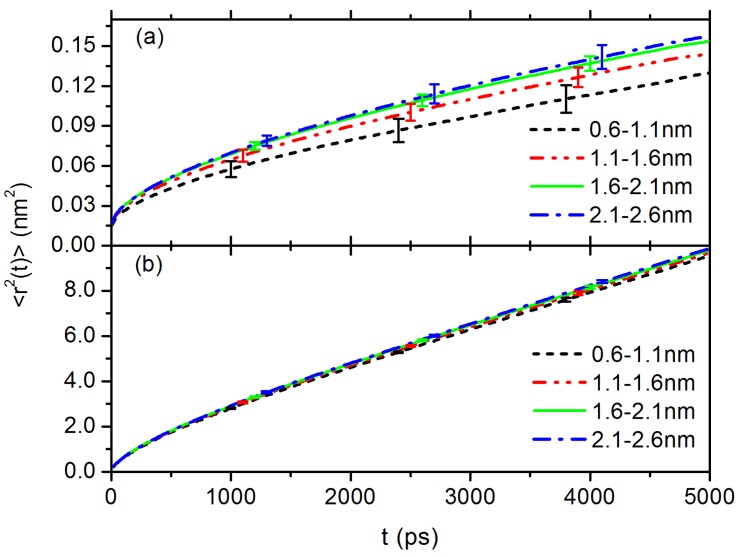
Layer resolved MSD of monomers in the case of: (**a**) PS around C${}_{60}$ NP; and (**b**) PP around C${}_{60}$ NP.

Presence of the C${}_{60}$ NP will influence the dynamics of the surrounding polymer segment, especially on the rotational dynamics of surrounding phenyl rings due to their parallel alignment on NP surface, as discussed in Figure 2a. To characterise such influences, we calculate the layer resolved time auto-correlation function (ACF) of the normal vector of the surrounding phenyl rings and the ACF of the carbon bond connecting phenyl ring and the PS chain backbone, at given distances to the centre of mass (CM) of the C${}_{60}$ NP. The results are shown in Figure 3a,b, respectively. Because both C${}_{60}$ NP and styrene monomers are moving, surrounding styrene monomers are frequently switching between different layers. Therefore, in our calculation, ACF of each vector is partitioned into layers according to their residence time in each layer on a short time scale of 5 ns. In particular, we firstly cut the final 1.5 μs long trajectory into short ones (5 ns each), and then calculate ACF of each vector for each 5 ns long trajectory, the result is partitioned into layers according to its residence time in the corresponding layer. Thereafter, the average is done over these short trajectories. It shows that the rotational dynamics of phenyl rings surrounding C${}_{60}$ NP surface are largely hindered. Such slowing down effect is also observed in the translational diffusion of styrene monomers. Figure 4a is the layer resolved mean-square displacement (MSD, $<r^{2}\left(t\right)>$) of styrene monomers around C${}_{60}$ NP. Interestingly, both rotational dynamics (Figure 3) and translational diffusion (Figure 4a) converged to bulk behaviour at a distance of r=1.6 nm to the CM of C${}_{60}$ NP, which is consistent with the orientation of phenyl rings reported in Figure 2. However, we do not observe such slowing down effect in the case of PP, as shown in Figure 4b. Note that due to a lower density of the PP system, propylene monomers are overall much faster than styrene monomers.

### 3.2. Diffusion Dynamics of C${}_{60}$ NP

**Figure 5:**
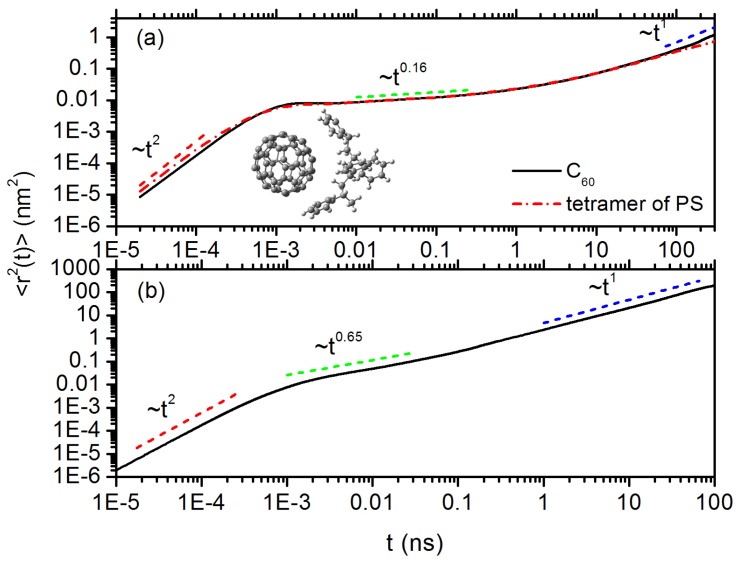
The centre-of-mass MSDs (solid lines) for C${}_{60}$ NP in: (**a**) PS melt; and (**b**) PP melt. The inset in (**a**) shows an example configuration where C${}_{60}$ NP is chelated by two phenyl rings on PS chain backbone. Dash dot line in (**a**) is the MSD of the tetramer on PS chain.

Figure 5a shows the results of MSD of C${}_{60}$ in PS melt, where we can see that the MSD typically spans three distinct regimes [62]: (i) short-time ballistic regime where $<r^{2}\left(t\right)>∼t^{2}$ before t= 6 ps; (ii) intermediate subdiffusive regime where $<r^{2}\left(t\right)>∼t^{0.16}$ in a time range of 6 ps <t< 70 ns; and (iii) long-time normal diffusive regime where $<r^{2}\left(t\right)>∼t^{1}$ after t> 70 ns. For the diffusion of C${}_{60}$ NP in PP melt (shown in Figure 5b), we also see three distinct regimes, however the time scaling exponent ν∼0.65 in subdiffusive regime is much larger than 0.16 in PS melt. Many studies have shown typical MSD behaviors in a confined environment, i.e., there is always a transition from a short-time subdiffusive regime to a long-time normal diffusive regime with a linear scaling of $<r^{2}\left(t\right)>∼t^{1}$ [63]. Another difference between two systems is that C${}_{60}$ diffuses much faster in Fickian regime in PP melt than in PS, the reason can be attributed to the fact that PP melt has a lower density than PS. Fast dynamics of probe C${}_{60}$ NP is also consistent with lower $T_{g}$ in PP system [12]. More importantly, we believe confinements induced by local interactions between C${}_{60}$ and phenyl rings play crucial roles, which is responsible especially for the small exponent of 0.16 in subdiffusive regime in PS melt. Interestingly, we find that a subsection of tetramer on PS chain has overall very similar MSD as C${}_{60}$ in the subdiffusive regime, as shown in Figure 5a, which indicates a dynamic coupling between C${}_{60}$ and tetramers on PS chain. Indeed, we find many configurations where C${}_{60}$ NP is chelated by two phenyl rings from such tetramer groups and these two phenyl rings are usually separated by ≤2 styrene monomers along PS chain backbone. These two phenyl rings are aligned parallel on the NP surface. An example of such configuration is shown in the inset of Figure 5a, such chelation can promote the caging effect on dynamics of C${}_{60}$ in PS melt. It should be noted that such chelated structure between C${}_{60}$ and neighbouring phenyl rings could be also influenced by stereoisomerism or the alignment of phenyl rings on the PS chain backbone. In addition, size match between nanoparticle and tetramers also plays a key role for such coupling.

Usually, the distribution of the NP displacement is expected to be Gaussian when the diffusion is Fickian. At the same time, there is a consensus that a non-Gaussian distribution would lead to a non-Fickian diffusion at corresponding spatiotemporal scales. However, many studies have reported that, although the DDF deviates from Gaussian, the diffusion process can still be Fickian with $\langle r^{2}\left(t\right)\rangle ∼t$ at long time scales [64,65,66]. For instance, Wang et al. [66] showed that, for the diffusion of colloid particle on phospholipid bilayer tubules or in entangled biofilament networks, instead of following a Gaussian distribution, there was a long exponential tail at large distance in DDF even after the diffusion enters Fickian. Such non-Gaussian yet Fickian diffusion has also been observed in various systems [67,68,69], such as Brownian motion in supercooled liquids, and colloids in entangled actin suspensions. The DDFs can be calculated from the self-part of the van Hove function (VHF):

(1) $$G_{s}\left(r,\Delta t\right)={\langle \delta \left[r-(|r\left(t_{0}+\Delta t\right)-r\left(t_{0}\right)|)\right]\rangle }_{t_{0}}.$$

**Figure 6:**
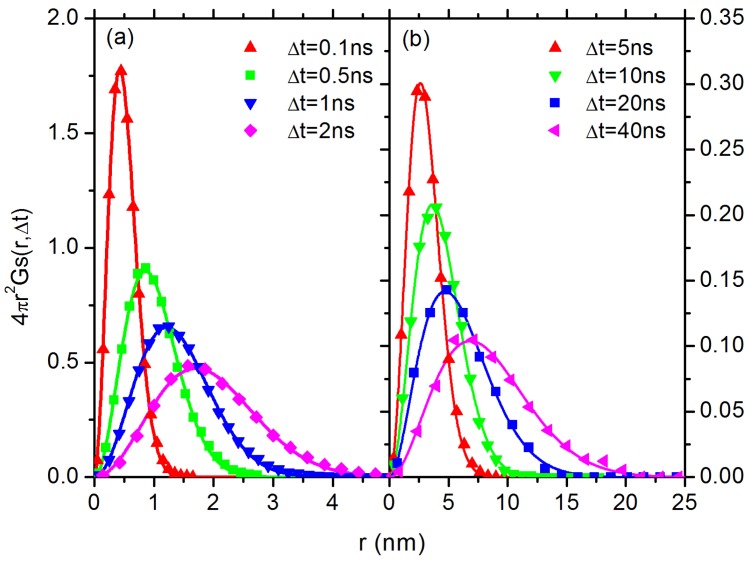
The DDF, $4\pi r^{2}G_{s}\left(r,\Delta t\right)$, for C${}_{60}$ in PP melt at different time scales (symbols): (**a**) the results of C${}_{60}$ in the subdiffusive regime at short time scale; and (**b**) the results of C${}_{60}$ in the normal diffusive regime at long time scale. The solid lines represent the corresponding Gaussian-fitting.

**Figure 7:**
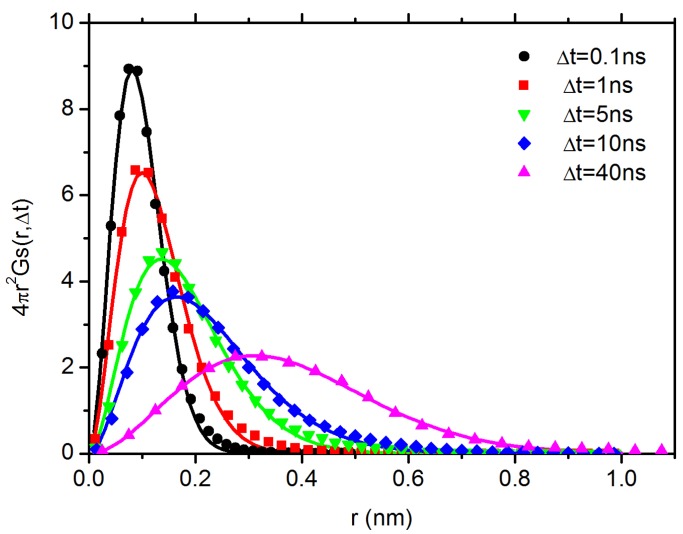
The DDF, $4\pi r^{2}G_{s}\left(r,\Delta t\right)$, for C${}_{60}$ in PS melt in the subdiffusive regime (symbols). The solid lines represent the corresponding Gaussian-fitting.

**Figure 8:**
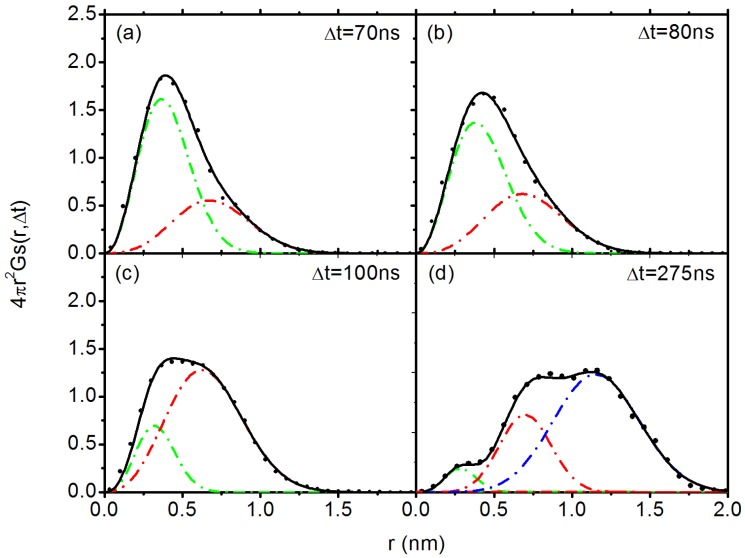
The DDF, $4\pi r^{2}G_{s}\left(r,\Delta t\right)$, for C${}_{60}$ in PS melt at different time scales (symbols). The dash dot lines are multiple Gaussian fittings, their sum is represented by the black solid line.

After multiplying a factor $4\pi r^{2}$, the product $4\pi r^{2}G_{s}\left(r,\Delta t\right)$ denotes the probability of a particle to be found at a distance *r* after a given time interval Δt. Note that diffusion of C${}_{60}$ has entered Fickian in both systems in the simulation time range. The results of DDFs for C${}_{60}$ in PP system in the short-time subdiffusive regime and long-time normal diffusive regime are shown in Figure 6a,b, respectively. It can be seen that the $G_{s}\left(r,\Delta t\right)$ curve for C${}_{60}$ in PP system can be easily fitted with Gaussian function on different time scales, ranging from 0.1 ns in subdiffusive regime to 40 ns in Fickian regime. The results of C${}_{60}$ in PS system in the subdiffusive regime and normal diffusive regime are shown in Figure 7 and Figure 8, respectively. In the subdiffusive regime, the $G_{s}\left(r,\Delta t\right)$ curve of C${}_{60}$ in PS system (Figure 7) can also be easily fitted with a Gaussian function on different time scales. However, in the normal diffusive regime, we find multiple peaks in the DDF curves (Figure 8), which can be attributed to C${}_{60}$ hopping motion. It means that C${}_{60}$ NP can hop in space to break the confinement locally [36,70,71] formed in PS melt. In particular, the area of the secondary local peak in the DDF (Figure 8) becomes larger as the interval time Δt increases, indicating that it can perform hopping motion more easily on a relatively long time scale. The secondary local peak is located at ∼0.7 nm, which defines the cage size. With a further increase in Δt, we find appearance of a third peak at even larger distance; an example is shown in Figure 8d at 275 ns, which is the signal for the hopping from initial cage to the neighbouring one. Note that, although the curves in Figure 8a,b at 70 ns and 80 ns have similar shapes to those in Figure 6 and Figure 7, a single-Gaussian fitting does not match with the data, as shown in Figure S2. However, these data can be fitted very well with two-Gaussians, as shown in Figure 8a,b.

The difference in the diffusion dynamics of C${}_{60}$ in PS and PP melts can be attributed to different local interactions in two system, especially in the interface area, as have been discussed above. Similar phenomena have also been observed in various systems, for instance, Patti [45] also observed a non-Gaussian diffusion behavior of NP in polymer melts in molecular dynamics simulations using general Lennard–Jones type potentials. In our unentangled PS system, the C${}_{60}$ NP is trapped in the cages formed by surrounding phenyl rings aligned parallel to the NP surface; once the C${}_{60}$ NP escapes from the cage, it can perform hopping motion. Note that such hopping motion has also been observed in many other systems [72,73,74,75], such as liquid crystals, supercooled liquids, and glasses.

**Figure 9:**
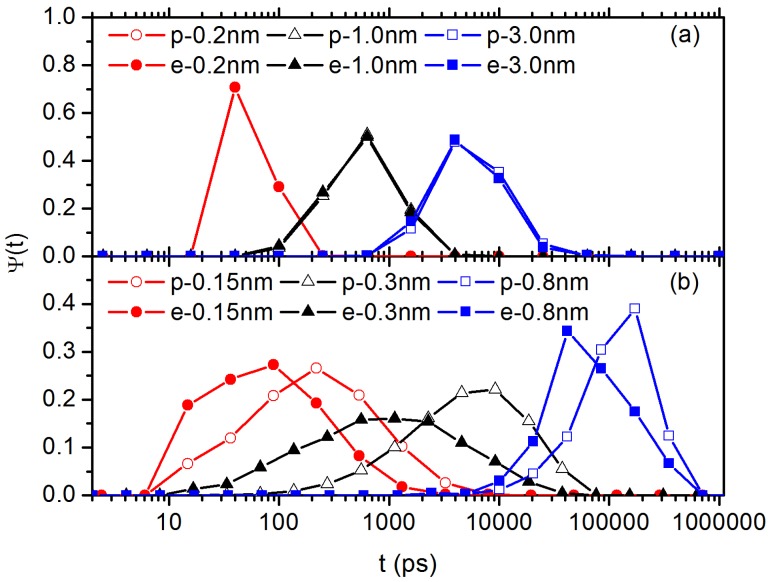
Distributions of exchange and of persistence times at various cutoff distances *d* for C${}_{60}$ NP in: (**a**) PP melt; and (**b**) PS melt. Symbols “p” and “e” in legend represent persistence time and exchange time, respectively.

To shed more light on the heterogeneous hopping motion of C${}_{60}$ in PS system, non-Gaussian parameter $\alpha_{2}$ (see Equation (2) in SM) is calculated to indicate the extent of deviation from Gaussian of the NP dynamics. Detailed results can be found in Figure S3. This parameter has a very small value in PP melt, indicating a homogeneous Gaussian process. While in the PS melt, its value increases with time at short time scale, while such deviation is not very significant since $\alpha_{2}$ only has a maximum value of ∼0.6. In addition, we calculate the distributions of so-called “persistence” time and “exchange” time. These concepts have been proven useful in explaining intermittent and heterogeneous dynamics [76,77]. Their definitions are as follows: If we define events that a particle diffuses beyond a given cutoff length *d*, then we record the time series when these events occur for each particle. Assuming a particle *i*, the initial position is $r_{i}\left(0\right)$, the first event time for this particle is $t_{1}$, namely $|r_{i}\left(t_{1}\right)-r_{i}\left(0\right)|=d$. The second event takes place at time $t=t_{1}+t_{2}$, that is $|r_{i}\left(t_{1}+t_{2}\right)-r_{i}\left(t_{1}\right)|=d$. The third event occurs after a further waiting time $t_{3}$ and so on. Then, we list all the waiting times between these events $\left\{t_{1},t_{2},t_{3},\cdots \right\}$. Moreover, the time $t_{1}$ has a different physical meaning from $t_{2}$, $t_{3}$, ⋯. The time $t_{1}$ is the time for the first event to occur which does not depend on when the previous event occurred, while the times $t_{2}$, $t_{3}$, ⋯ are times for the following consequent events. Thus, we address the time $t_{1}$ as persistence time, and the times $t_{2}$, $t_{3}$, ⋯ as exchange time. The distributions of the persistence time and exchange time of C${}_{60}$ in PP and PS systems are shown in Figure 9a,b, respectively. In PP melt, the distributions of exchange and persistence times are coincident with each other, indicating that the dynamics of C${}_{60}$ NP in PP system is homogeneous. In Figure 9b, in PS melt, we find clear difference between distributions of the exchange and persistence time, and the persistence time is much larger than the exchange time, which is in agreement with previous simulations [77]. These results demonstrate that the dynamics of the C${}_{60}$ NP is significantly heterogeneous in PS system, consistent with the hopping motion of C${}_{60}$ NP. It also indicates that consequent events can occur more likely if the first event has just occurred, which has been observed widely in supercooled glass formers [77,78].

**Figure 10:**
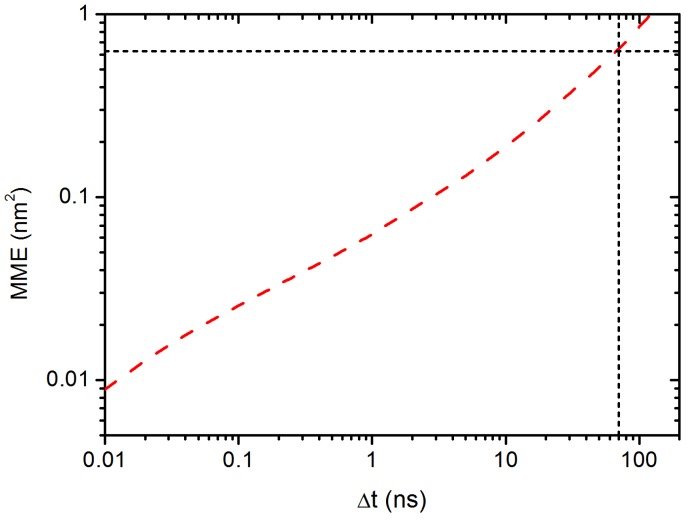
The second moment of MME as a function of time Δt for C${}_{60}$ in PS melt.

**Figure 11:**
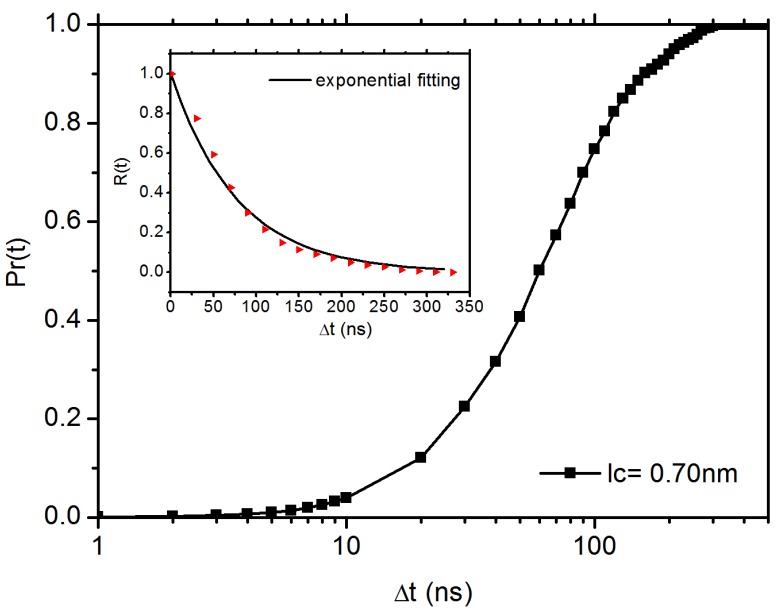
The probability (Pr(t)) for the C${}_{60}$ NP to escape from the cage with a radius lc=0.7 nm. The inset shows the probability for the C${}_{60}$ NP to be maintained in the cage *R*(t) = 1 −Pr(t).

Furthermore, we calculate the residence time of C${}_{60}$ NP in a transient cage formed by its neighbouring phenyl rings, which is defined as the time duration for which the NP remains in cage. The cage size is defined with a radius of $l_{c}$ = 0.7 nm, where the average orientation angle (shown in Figure 2a) of the phenyl ring < 15${}^{\circ }$. $l_{c}$ is also the position where we find the appearance of the secondary peak in DDF, as shown in Figure 8. When the mean maximal excursion (MME) distance of the C${}_{60}$ NP is larger than the cage radius $l_{c}$, the C${}_{60}$ NP is considered to have broken through the cage. Here, the mean maximal excursion distance ($r_{max}$) is defined as $r_{max}\left(t\right)=max\left\{r\left(t^{\prime }\right):0\leq t^{\prime }\leq t\right\}$; we plot the result of MME in Figure 10. The probability for the C${}_{60}$ NP to escape from the cage Pr(t) = 1 if $r_{max}\geq l_{c}$, otherwise Pr(t)=0 [79]. The result of Pr(t) is shown in Figure 11. We can see that the C${}_{60}$ NP has a probability of 40% to escape from the cage over 50 ns. Therefore, equation $R\left(t\right)=1-Pr\left(t\right)∼e^{-t/\tau }$ can be used to characterise the probability of the C${}_{60}$ NP to be maintained in the cage after *t*, τ is the mean residence time of the C${}_{60}$ NP in the cage. As pointed out by Volgin et al. [44], this algorithm does not consider the case that the C${}_{60}$ NP jumps back into the cage. However, they are very rare events, therefore the probability of reversible escape is not critical for our result. The result for R(t) probability is shown in the inset of Figure 11. By a least squares fitting with equation $R\left(t\right)∼e^{-t/\tau }$, we obtain τ=77 ns. It is worth mentioning that the value τ is consistent with the time which marks the onset of the Fickian region on MSD curve (Figure 5). We also note that at this timescale, the mean maximal excursion distance that the C${}_{60}$ NP can diffuse is ≤0.8 nm, as indicated by a horizontal dash line in Figure 10, which is also consistent with the $l_{c}$ we used. Hence, the results of residence time provide another good evidence for C${}_{60}$ NP hopping in PS melt. The exponential distribution of the residence time in the inset of Figure 11 also indicates a typical Poisson process with uncorrelated, random hopping of C${}_{60}$ NP.

**Figure 12:**
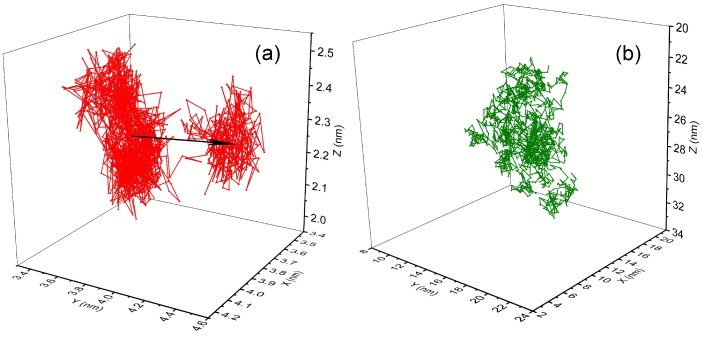
The 150 ns trajectory of fullerene in: (**a**) PS melt; and (**b**) PP melt. The arrow indicates a jump motion.

Finally, we also plot the trace lines of C${}_{60}$ NP in PS and PP systems during a 150 ns long trajectory; step size is set at Δt = 0.1 ns between frames, as shown in Figure 12a,b, respectively. In PS system, we can observe that C${}_{60}$ NP motion is confined in two discrete localised regions (see Figure 12a). In between, the two regions are connected by a single trajectory trace line, which can be regarded as a jump consisted of several successive steps, as indicated by an arrow in the figure. In contrast, we do not observe any such jumps in PP system (see Figure 12b): the dynamics of C${}_{60}$ NP in PP system seems to be more homogeneous. Thus, considering the DDF of C${}_{60}$ NP in PS system in normal diffusive regime (Figure 8), the displacements at shorter distance correspond to the motion in local confined areas, whereas the secondary peak at longer distance can be attributed to hopping motion. The results provide straightforward evidence for the existence of the hopping motion of C${}_{60}$ NP in PS melt. Note that, due to much faster diffusion of C${}_{60}$ in PP melt, length scale in Figure 12b is much larger than that in Figure 12a, although these two figures are plotted at the same time scale. For comparison, representative results of C${}_{60}$ NP in PP system on a similar length scale as that in Figure 12a are shown in Figure S4. We still do not observe any jump-like motion in these figures.

## 4. Conclusions

By performing all-atom molecular dynamics simulations, we investigate the diffusion dynamics of C${}_{60}$ NP in two linear polyvinyl polymers, namely PS and PP melts. At the same time, the interfacial structure and dynamics properties of polymer segments around C${}_{60}$ NP surface in both composite systems are also investigated. In the case of PS, due to attractive interactions between C${}_{60}$ and phenyl ring side groups on chain backbone, phenyl rings are found to adopt a parallel configuration on the C${}_{60}$ NP surface. We also find a dynamic coupling between C${}_{60}$ NP and tetramer segment on PS chain backbone which is commensurate with C${}_{60}$ in size. Such dynamic coupling comes from a chelation effect formed by parallel aligned phenyl rings of such tetramer segment on NP surface. In return, C${}_{60}$ has a slow down effect on both rotational and translational diffusion of surrounding styrene monomers. Influence of C${}_{60}$ on static orientation, translational and rotational diffusion of phenyl rings are found to occur at the same distance range. However, in the case of PP, C${}_{60}$ has almost no influences on the dynamics of surrounding monomers.

From the MSD results of NP in both polymer melts, we find that the NP diffusion spans three distinctive regimes: short-time ballistic (α=2), intermediate subdiffusive (0<α<1), and long-time Fickian diffusive (α=1) regions. In the normal diffusive regime, our results show that the DDF of C${}_{60}$ NP follows a Gaussian form in PP system. However, the DDF of C${}_{60}$ NP in PS melt can be decomposed into several Gaussians, which indicates the existence of NP hopping. The difference between the two systems can be attributed to the specific interaction between C${}_{60}$ and phenyl rings on PS chain backbone. The distributions of exchange time and persistence time suggest that the diffusion dynamics of C${}_{60}$ NP in PP system is rather homogeneous, while, in PS system, the dynamics of the C${}_{60}$ NP is significantly heterogeneous due to its hopping motion. Moreover, we calculate the mean residence time τ of the C${}_{60}$ NP confined in a transient cage with a radius of 0.7 nm, formed by neighbouring phenyl rings parallel aligned on NP surface. The value of τ is consistent with the time which marks the onset of the Fickian diffusive regime. C${}_{60}$ NP can perform a jump-like motion as its displacement exceeds the cage size, i.e., 0.7 nm, as also indicated by the appearance of the secondary peak in the DDF. By monitoring the centre-of-mass trajectory of C${}_{60}$ NP in space in PS melt, we also observe that NP motion is confined in localised regions. These regions are connected by a jump-like motion composed of several successive steps. These results provide direct evidence for the existence of NP hopping. In contrast, the motion of C${}_{60}$ NP in PP system is more homogeneous.

In summary, we find a hopping motion of C${}_{60}$ NP in PS melt where NP has attractive interaction with phenyl ring side groups, which is similar to the results reported in Ref. [44]. According to both statistic dynamic theory and scaling theory, hopping is relevant only for particles with size $2R_{a}/d_{T}∼$ 1.5–2 where NP motion can be influenced by fluctuations of entanglement mesh, while smaller particles are considered to slip through the entanglement mesh. However, our results suggest that, due to local attractive interactions between NP and polymer segments, NP hopping can also happen on the length scale of its own size, which is much smaller than $d_{T}$. A well-defined interaction minimum between NP and polymer segment, as we found here a chelation effect on C${}_{60}$ NP surface by neighbouring phenyl rings on PS tetramer group, is the key for the NP hopping. Since C${}_{60}$ size (0.7 nm in diameter) is much smaller than $d_{T}$, investigations of diffusion dynamics of NPs that have locally attractive interaction with polymer segments on a length scale between $R_{a}$ and $d_{T}$ will be a possible direction of future work.

## Figures and Tables

**Table 1:** Characteristic properties of the equilibrated systems at simulated temperature *T* = 500 K.

	Box Size (nm)	Density (g/cm${}^{3}$)	$N_{\mathrm{chains}}$	$N_{\mathrm{length}}$	$R_{g}$ (nm)	$R_{\mathrm{ee}}$ (nm)	$\tau_{\mathrm{KWW}}$ (ns)
PS	5.27	0.952	40	20	0.982 ± 0.006	2.566 ± 0.124	792.03
PP	5.18	0.715	70	20	0.836 ± 0.017	2.041 ± 0.085	0.86

## Supplementary Materials

These are available at http://www.mdpi.com/2073-4360/10/6/598/s1.
