# Supplementary material for: Interfacial Properties and Hopping Diffusion of Small Nanoparticle in Polymer/Nanoparticle Composite with Attractive Interaction on Side Group
Source: Polymers (Basel). 2018 May 29;10(6):598. doi: 10.3390/polym10060598 (PMC6403981; doi:10.3390/polym10060598)
Supplement: Supplementary file 1 [file polymers-10-00598-s001.pdf]

Supplementary Information

Interfacial Properties and Hopping Diffusion of Small Nanoparticle in Polymer/Nanoparticle Composite with Attractive Interaction on Side Group

Kai-Xin Ren [1],Xiang-Meng Jia[1], Gui-Sheng Jiao[1,†], Tao Chen[1], Hu-Jun Qian [1*] and Zhong-Yuan Lu [1]

1 State Key Laboratory of Supramolecular Structure and Materials, and Laboratory of Theoretical and Computational Chemistry, Institute of Theoretical Chemistry, Jilin University, Changchun 130023, China

* Correspondence: hjqian@jlu.edu.cn; Tel.: +86-431-88498132

† Current address: China Spallation Neutron Source (CSNS), Institute of High Energy Physics (IHEP), Chinese Academy of Sciences (CAS), Dongguan 523803, China

Academic Editor: name

Version May 28, 2018 submitted to Polymers

1. Supplementary Information

We have calculated the time autocorrelation function (ACF) of the end-to-end vector of PS and PP chains to extract the relaxation time of the polymer chain. Data of ACF curves are shown in Fig. S1. The relaxation time τ of chains can be obtained by fitting the ACF curves with the Kohlrausch-Williams-Watts (KWW) function [1,2] as follows:

$$ACF(t) = Aexp\left[-(\frac{t}{\tau})\right]^{\beta} \quad (1)$$

Where A is a pre-exponential factor that can capture relaxation processes happening at very short times, the relaxation time that gives us an estimation of the characteristic decorrelation time of the end-to-end vector of the polymer chain, and β is an stretch exponent. The τ of the PS and PP chains are 792.03 ns and 0.86 ns, respectively.

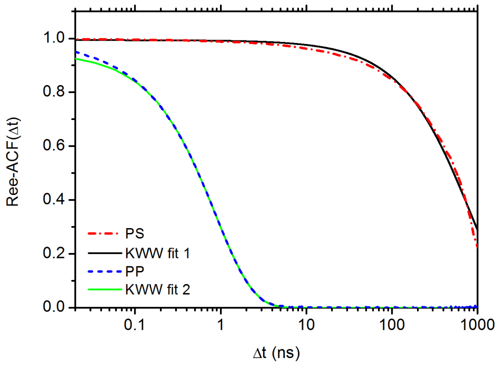

Fig. S1 The time autocorrelation function (ACF) of the end-to-end vector of PS and PP chains (dash lines). The solid lines represent the corresponding KWW-fitting.

Fig. S2 shows the fitting results of using one and two Gaussians to fit the DDF at time scale of 70ns for C_{60} in PS, we see that single-Gaussian fitting does not match well with the data points while two-Gaussians fitting works well.

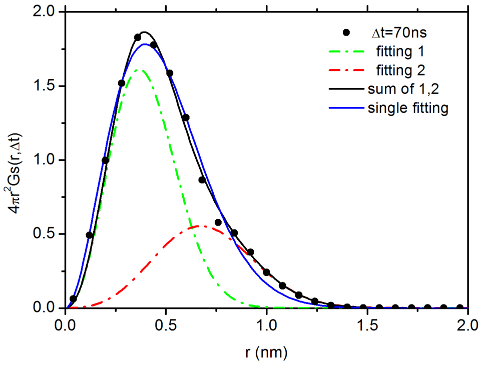

Fig. S2 The fitting results of using either single-Gaussing or two-Gaussians to fit the DDF at 70ns for C_{60} in PS.

In order to characterize the deviation from Gaussian, non-Gaussian parameter α_2 is calculated:

$$\alpha_2(\Delta t) = \frac{3\langle \Delta r^4(\Delta t)\rangle}{5\langle \Delta r^2(\Delta t)\rangle^2} - 1. \quad (2)$$

The results are shown in Fig. S3 for PS and PP systems. Generally, $\alpha_2 = 0$ indicates a perfect Gaussian process. As expected, this parameter has a very small value in PP melt, indicating a homogeneous Gaussian process. While when C_{60} NP diffuses in PS melt, its value increases with time indicating some deviations from Gaussian, while such deviation is not very significant since α_2 only has a maximum value of 0.6.

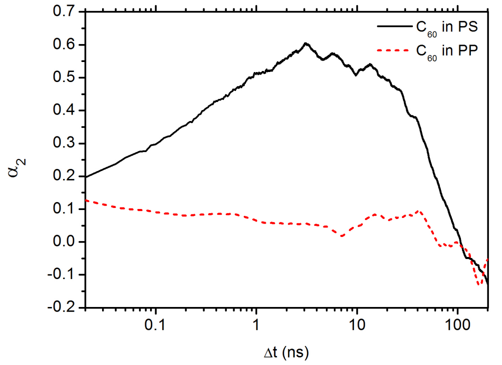

Fig. S3 The time dependant non-Gaussian parameter α_2 for the diffusion of C_{60} in PS melt (solid line) and PP melt (dash line).

The trajectory of C_{60} NP in PP system on a small length scale are shown in Fig. S4. And from the results we still do not observe any jump like motion of C_{60} in these figures.

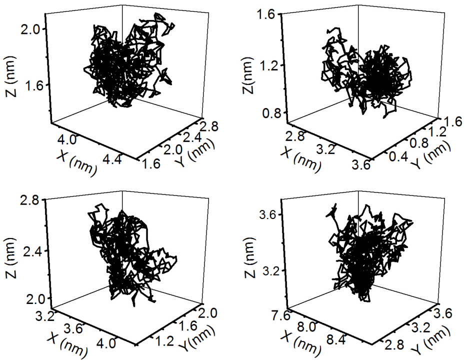

Fig. S4 The trajectory of C_{60} NP in PP system on a small length scale.

References

1. He, Y.; Lutz, T.R.; Ediger, M.D.; Ayyagari, C.; Bedrov, D.; Smith, G.D. NMR Experiments and Molecular Dynamics Simulations of the Segmental Dynamics of Polystyrene. *Macromolecules* **2004**, *37*, 5032–5039.
2. Harmandaris, V.A.; Floudas, G.; Kremer, K. Temperature and Pressure Dependence of Polystyrene Dynamics through Molecular Dynamics Simulations and Experiments. *Macromolecules* **2011**, *44*, 393–402.

© 2018 by the authors. Submitted to *Polymers* for possible open access publication under the terms and conditions of the Creative Commons Attribution (CC BY) license (http://creativecommons.org/licenses/by/4.0/).
